# Molar Pregnancy–Induced Hyperthyroidism: The Importance of Early Recognition and Timely Preoperative Management

**DOI:** 10.1210/jcemcr/luad129

**Published:** 2023-12-06

**Authors:** Laurel Walfish, Nisha Gupta, Dong Bach Nguyen, Mark Sherman

**Affiliations:** Department of Internal Medicine, McGill University, Montreal, Quebec H4A 3J1, Canada; Department of Endocrinology, McGill University, Montreal, Quebec H4A 3J1, Canada; Department of Obstetrics and Gynecology, McGill University Health Center, Montreal, Quebec H4A 3J1, Canada; Department of Endocrinology, McGill University Health Centre, Montreal, Quebec H4A 3J1, Canada

**Keywords:** molar pregnancy, hyperthyroidism, perioperative management

## Abstract

Hyperthyroidism due to gestational trophoblastic disease (GTD) is a rare but potentially life-threatening condition. Optimal perioperative management is crucial for favorable outcomes and prevention of thyroid storm. However, scarce data exist defining the ideal approach to this complex clinical presentation. This case report describes a first-time pregnant 32-year-old woman who was found to be biochemically hyperthyroid in the context of a 10-week gestation molar pregnancy. Despite her biochemical values, the patient remained clinically asymptomatic of her thyroid disease. The Gynecology and Anesthesiology services urgently consulted Endocrinology, and empiric treatment for prevention of potential impending thyroid storm was initiated prior to operative uterine evacuation. After 2 uneventful dilation and curettages with chemotherapy and a transient prescription of antithyroid medication, the patient normalized her human chorionic gonadotropin (hCG) level and recovered to biochemical euthyroidism. Other than a pruritic rash that may have been due to propylthiouracil, the patient's hyperthyroidism improved without further complications. This case highlights the importance of recognizing the link between GTD and thyrotoxicosis to allow for timely initiation of appropriate preoperative treatment. Fortunately, the multidisciplinary approach facilitated management to prevent evolution to thyroid storm.

## Introduction

Gestational trophoblastic disease (GTD) is an unregulated proliferation of placental trophoblastic tissue and is an obstetrical emergency affecting about 1 per 1000 pregnancies [1]. In a typical pregnancy, β-human chorionic gonadotropin (hCG) plays a weak thyrotrophic role that occasionally causes transient gestational hyperthyroidism [2]. In GTD, significantly elevated hCG levels have the potential to further increase the production of thyroid hormone, leading to thyrotoxicosis. Early preoperative detection and management through a multidisciplinary approach is paramount to avoid thyroid storm. However, scarce data have been reported suggesting the optimal treatment, as GTD-induced hyperthyroidism is a rare clinical entity. This case report describes our management of a woman with GTD and the multidisciplinary efforts taken to safely prevent evolution to thyroid storm.

## Case Presentation

A 32-year-old woman, in her first pregnancy, at 10 weeks of gestation presented to medical care with a 2-week history of bloating, abdominal pain, and nausea. She denied bleeding or any symptoms of thyroid dysfunction. She had no significant past medical history, and her family history was unremarkable for any thyroid disorders.

## Diagnostic Assessment

On examination, she was afebrile with blood pressure of 115/67 mm Hg and heart rate of 88 beats per minute. Head and neck examination demonstrated a non-tender and normal sized thyroid. There was no evidence of ophthalmopathy, acropachy, pretibial myxedema, or tremor. The remainder of the general examination was unremarkable.

**Figure 1.**
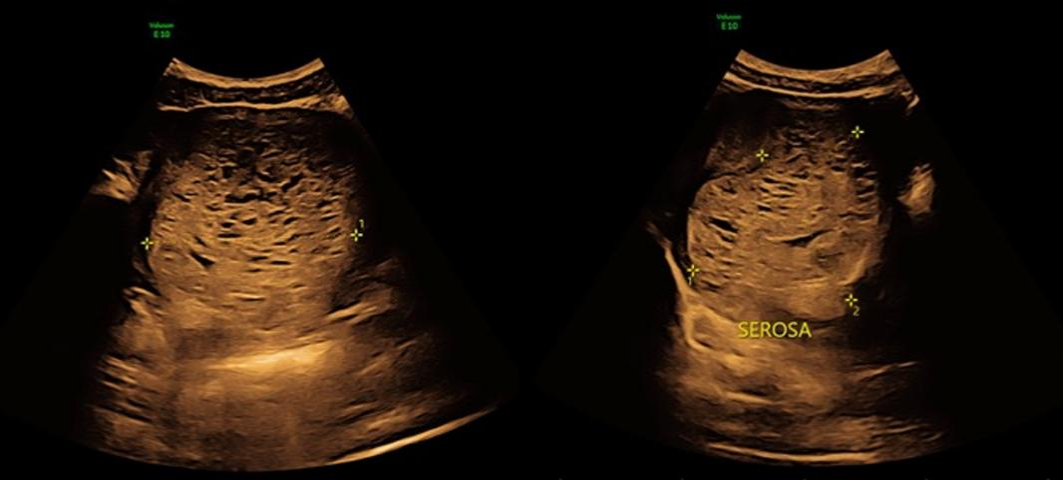
Ultrasound image of the 10-cm molar pregnancy invading the myometrium.

**Figure 2.**
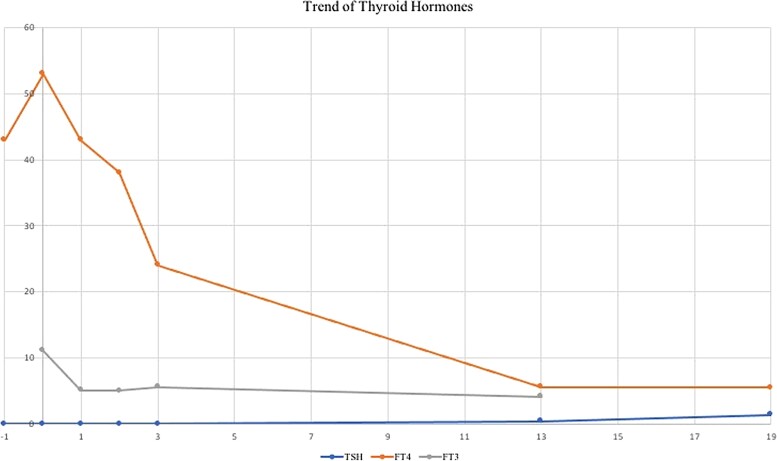
Trend of thyroid hormones. Thyroid stimulating hormone (TSH; reference range, 0.40-4.40 mIU/L, 1 mIU/L = 1 µIU/mL); free thyroxine (FT4; reference range 8-18 pmol/L, 1 pmol/L = 0.0775 ng/dL); free triiodothyronine (FT3; reference range 3.8-6.0 pmol/L, 1 nmol/L = 64.9 ng/dL). X axis represents day since surgery.

Laboratory findings demonstrated a hCG of over 420 million IUs/L (normal hCG between 8 and 10 weeks gestation is 60 000-90 000 IUs/L) (420 million mIU/mL) [3]. Pelvic ultrasound was suspicious for a larger than 10-cm molar pregnancy that appeared to invade the posterior myometrium (Fig. 1). Her thyroid profile was consistent with overt biochemical hyperthyroidism with an undetectable thyroid stimulating hormone (TSH; normal range, 0.4-4.4 pmol/L) and free thyroxine (T4) of 43.1 pmol/L (3.34 ng/dL) (normal T4 range, 8.0−18.0 pmol/L) (Fig. 2). Free triiodothyronine (T3) levels and thyroid receptor antibody (TRAb) were not initially obtained.

## Treatment

Upon discovery of her suspected invasive mole and biochemical hyperthyroidism, the patient was transferred to our quaternary care center for multidisciplinary specialist evaluation. A diagnostic laparoscopy with uterine evacuation under ultrasound guidance was promptly scheduled. The treating team urgently consulted the Endocrinology service, given the concern for potential impending thyroid storm with the upcoming surgery. The patient was started on intravenous (IV) corticosteroids (hydrocortisone 100 mg IV once and then 50 mg IV every 8 hours) and an antithyroid agent (propylthiouracil [PTU] 200 mg per os [PO] every 4 hours), while rapid acting beta blockers remained on standby. The procedure occurred as planned, without intra-operative evidence of posterior uterine wall invasion, and she was empirically admitted to the intensive care unit postoperatively for observation, where she remained clinically stable. After 24 hours, her PTU was reduced to 200 mg PO twice daily and her corticosteroid therapy was discontinued. Considering the high initial hCG level on presentation, the gynecology service elected to administer one dose of Actinomycin-D with dexamethasone as chemoprophylaxis for the development of gestational trophoblastic neoplasia. The patient was discharged in stable condition from the hospital on postoperative day 3 with PTU 200 mg twice daily to be continued as an outpatient.

## Outcome and Follow-Up

**Figure 3.**
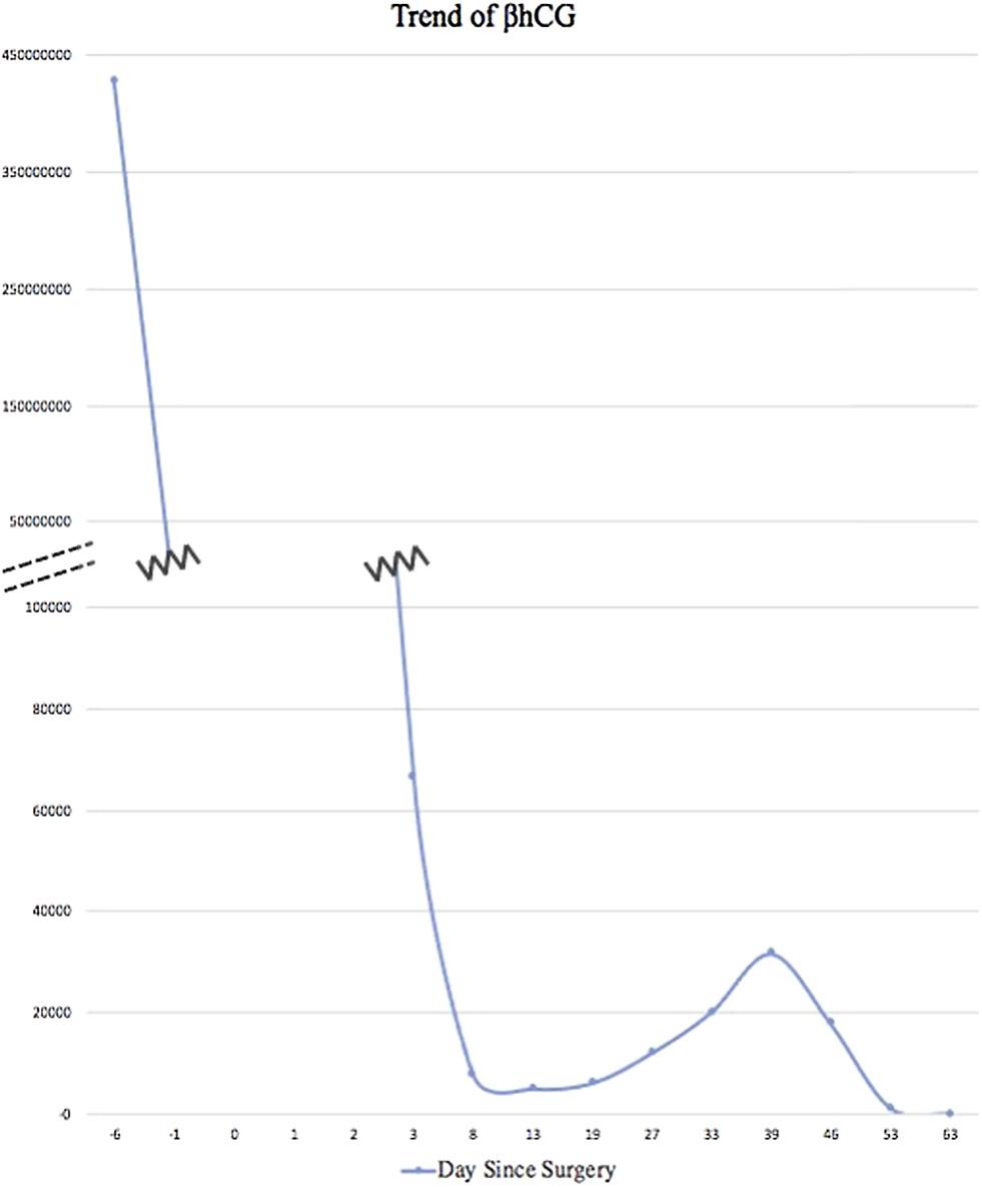
Trend of β-human chorionic gonadotropin (β-hCG; reference range 0.0-4.9 IUs/L, 1 IU/L = 1 mIUmL).

Prompt follow-up appointments were organized with Endocrinology and Gynecology for close hCG and thyroid hormone monitoring. Within 2 weeks of the procedure, blood tests demonstrated resolution of the patient's biochemical hyperthyroid status and correlated with declining hCG levels (Figs. 2 and 3). Given that the patient was clinically euthyroid and newly biochemically hypothyroid with a FT4 level of 5.6 pmol/L (0.434 ng/dL), PTU was discontinued on postoperative day 14 (Fig. 2). Further prompting its cessation, the patient had developed a pruritic rash that may have been associated with the antithyroid agent. Nineteen days postoperation, while thyroid function remained unchanged, her hCG levels started to increase (Fig. 3). The final surgical pathology had shown complete hydatidiform mole, yet repeat imaging was suggestive of recurrent invasion. As the hCG levels continued to rise, the patient underwent a second dilation and curettage in combination with 7 cycles of Methotrexate chemotherapy regimen. Thyroid hormone levels remained stable despite the fluctuations of hCG, and the patient showed no evidence of metastatic disease. Following the second procedure and chemotherapy treatment, the patient's hCG decreased post treatment and continued to remain normal several months later, as did her thyroid function (Figs. 2 and 3).

## Discussion

In this report, we present the case of a 32-year-old woman with molar pregnancy–induced hyperthyroidism. This rare clinical entity is mediated by elevated levels of hCG, produced by the mole, that stimulate TSH receptors. In general, the hCG hormone is thought to have a potency of 1 in 4000 compared to TSH itself and therefore only very high levels have clinically significant thyroid effects [4]. However, in GTD, the hCG hormone is considered to be more stimulating than the hormone produced by normal pregnancy [4]. Levels of hCG above 100 000 and persistently elevated for several weeks are considered risk factors for thyrotoxicosis [4, 5]. For these reasons, the National Comprehensive Cancer Network recommends thyroid function tests to be performed in all patients with a molar pregnancy [1]. Our patient's extremely increased hCG level, measured at over 420 million IUs/L, had the capability to significantly stimulate her thyroid hormone production. Yet despite this high value, she remained clinically asymptomatic of her thyroid disease.

Features of GTD are shared with other hCG-induced hyperthyroid states, the most frequent being gestational transient thyrotoxicosis (GTT). GTT is present in about 1% to 3% of all pregnancies and is usually limited to the first half of gestation [6]. The typical biochemical findings include elevated hCG, undetectable TSH, elevated T4, infrequently elevated T3, and the absence of thyroid receptor antibodies. Our patient's laboratory findings were consistent with this described pattern. Furthermore, comparable to how our patient presented, patients with GTT present with hyperemesis gravidarum from the increased hCG levels but rarely have overt clinical symptoms of hyperthyroidism [2, 6, 7]. Typical symptoms of hyperthyroidism during gestation can include failure to gain weight, heat intolerance, excessive sweating, and tachycardia; however, this can sometimes be difficult to differentiate from physiologic changes in pregnancy [7].

Typically, hCG-induced hyperthyroidism does not require treatment, since the condition resolves when hCG levels decrease through pregnancy [2]. In contrast, with a molar pregnancy, a return to a biochemical euthyroid state requires surgical uterine evacuation and medical treatment to achieve proper reduction in hCG levels. It is therefore of utmost importance to recognize the link between GTD and thyrotoxicosis and to initiate appropriate preoperative treatment of hyperthyroidism as soon as possible.

Women with GTD-associated hyperthyroidism require pre-emptive medical management of the thyrotoxicosis prior to interventions, as severe consequences can occur if left untreated [7]. Complications can include extreme hyperthermia, tachycardia, hypertension, coma, or death [7, 8]. In general, thyroid storm has a mortality of 10% to 20% and therefore must be managed with extreme caution. In patients undergoing surgery, thyroid storm can be triggered by the surgical stress of the operation or the anesthesia itself [7]. Ideally, patients should wait until they are euthyroid prior to undergoing surgery, although euthyroidism typically takes weeks to occur. In the case of our patient, surgery was a necessary therapeutic measure to eliminate the inciting agent and was hence performed without delay.

There are currently no evidence-based recommendations for the management of GTD-induced hyperthyroidism. The 2016 American Thyroid Association (ATA) Guidelines recommend treatment of hyperthyroidism due to choriocarcinoma, a similar cancerous variation of GTD, to include both methimazole and treatment directed against the primary tumor (strong recommendation, low quality evidence) [7]. Our patient underwent both of these treatments processes; however, further specifics of management were left to clinical judgment in the absence of accompanying recommendations. Therefore, a multidisciplinary approach including physicians from Obstetrics and Gynecology, Endocrinology, Anesthesia, and Critical Care facilitated optimal management for our patient and prevented complications.

We opted to treat our patient with high dose steroids and PTU prior to her surgical intervention. High doses of intravenous steroids functioned to reduce the T4 to T3 conversion [8]. PTU was used over methimazole for its faster action of decreasing circulating T3 levels, given the urgency of the situation. With this management and after 24 hours of observation in the intensive care unit postoperation, the patient was hemodynamically stable without evidence of clinical thyrotoxicosis and the treatment was therefore slowly and successfully weaned.

Our management was comparable to other documented case reports in the literature. Blick et al and De Guzman et al both describe cases of patients presenting with molar pregnancies already in thyrotoxicosis or thyroid storm. Their patients were treated with high dose steroids, PTU, propranolol, and definitive management of evacuation of molar pregnancies which resulted in improvement of thyroid disease [5, 9]. In contrast, our patient never showed symptoms of hyperthyroidism and our treatment was empirical. Scarce reports in the literature were found summarizing the approach to clinical management when the patient was not clinically in thyrotoxicosis, such as our patient. Nevertheless, our precautions were crucial to safely evacuate our patient's molar pregnancy. Further preventative measures could have also been taken in terms of choosing the optimal anesthetic during the operation. General anesthesia is less favored over regional anesthesia in the thyrotoxic patient; however, it was unavoidable in our patient undergoing an exploratory laparoscopy [10].

In conclusion, this case highlights the importance of recognizing the link between GTD and thyrotoxicosis to allow for timely initiation of appropriate preoperative treatment. We do not know if our patient's situation would have devolved without our interventions, but the risk was sufficient to warrant our multidisciplinary approach, which facilitated management to prevent evolution to thyroid storm. Although our patient safely recovered from her GTD-induced hyperthyroid state, more research is needed to define the optimal treatment plan for these atypical presentations.

## Learning Points

- It is essential to recognize the link between GTD and thyrotoxicosis to allow for initiation of appropriate management as soon as possible.
- Women with GTD-associated hyperthyroidism require pre-emptive medical treatment of the anticipated thyrotoxicosis prior to interventions, as severe consequences can occur if left untreated.
- A multidisciplinary approach during the perioperative period can facilitate optimal management for the patient and prevent complications.

## Contributors

All authors made individual contributions to authorship. L.W. contributed to the drafting and revision of the manuscript. N.G. contributed to the clinical care of the patient and drafting and revision of the manuscript. D.B.N. contributed to the clinical care of the patient and revision of the manuscript. M.S. contributed to the clinical care of the patient and revision of the manuscript. All authors reviewed and approved the final draft.

## Data Availability

Data sharing is not applicable to this article as no datasets were generated or analyzed during the current study.

## Abbreviations

GTD: gestational trophoblastic disease
GTT: gestational transient thyrotoxicosis
hCG: human chorionic gonadotropin
IV: intravenous
PO: per os
PTU: propylthiouracil
T3: triiodothyronine
T4: thyroxine
TSH: thyrotropin (thyroid stimulating hormone)
